# Epigenetic Targeting of Autophagy via HDAC Inhibition in Tumor Cells: Role of p53

**DOI:** 10.3390/ijms19123952

**Published:** 2018-12-08

**Authors:** Maria Mrakovcic, Lauren Bohner, Marcel Hanisch, Leopold F. Fröhlich

**Affiliations:** Department of Cranio-Maxillofacial Surgery, University of Münster, Albert-Schweitzer-Campus 1, 48149 Münster, Germany; maria.mrakovcic@web.de (M.M.); lauren.oliveiralimabohner@ukmuenster.de (L.B.); marcel.hanisch@ukmuenster.de (M.H.)

**Keywords:** HDAC, HDACi, SAHA, autophagy, p53, apoptosis, tumor

## Abstract

Tumor development and progression is the consequence of genetic as well as epigenetic alterations of the cell. As part of the epigenetic regulatory system, histone acetyltransferases (HATs) and deacetylases (HDACs) drive the modification of histone as well as non-histone proteins. Derailed acetylation-mediated gene expression in cancer due to a delicate imbalance in HDAC expression can be reversed by histone deacetylase inhibitors (HDACi). Histone deacetylase inhibitors have far-reaching anticancer activities that include the induction of cell cycle arrest, the inhibition of angiogenesis, immunomodulatory responses, the inhibition of stress responses, increased generation of oxidative stress, activation of apoptosis, autophagy eliciting cell death, and even the regulation of non-coding RNA expression in malignant tumor cells. However, it remains an ongoing issue how tumor cells determine to respond to HDACi treatment by preferentially undergoing apoptosis or autophagy. In this review, we summarize HDACi-mediated mechanisms of action, particularly with respect to the induction of cell death. There is a keen interest in assessing suitable molecular factors allowing a prognosis of HDACi-mediated treatment. Addressing the results of our recent study, we highlight the role of p53 as a molecular switch driving HDACi-mediated cellular responses towards one of both types of cell death. These findings underline the importance to determine the mutational status of p53 for an effective outcome in HDACi-mediated tumor therapy.

## 1. Introduction: HDAC Inhibitors as Epigenetic Cancer Drugs

Cancer is an intensively investigated versatile complex disease resulting from genetic alterations that provoke constitutive activation of oncogenes or functional silencing of tumor suppressor genes [1,2,3]. At the cellular level, tumorigenesis is reflected by a multistep process characterized by cell death resistance, dysregulated intracellular growth signaling and metabolism, as well as sustained angiogenesis. In recent years, the focus of molecular cancer studies has been directed towards epigenetic analyses. In contrast to genetic mutations which are based on modifying DNA sequence, inherited abnormal epigenetic patterns additionally modify gene expression without alteration of the primary gene sequence and thereby integrate multiple levels of regulatory pathways. Epigenetic processes known to date include DNA methylation, histone modifications, and chromatin remodeling (i.e., restructuring of nucleosomes), but also modulation of gene expression via non-coding RNAs that have been added only recently [4,5,6,7]. Deviant epigenome with frequent aberrant DNA methylation patterns and histone modifications, for example hyper-methylation or hypo-acetylation of tumor suppressor genes has been defined in diverse tumor entities [8,9,10,11]. Among the histone modifications, predominantly acetylation or deacetylation, that are executed by different enzyme classes of histone acetyltransferases (HATs) and histone deacetylases (HDACs), respectively, have been thoroughly studied because of their crucial role in directing gene expression by altering the chromatin structure [12]. The physiological functions of HAT/HDAC have not been exhaustedly elucidated but involve key cellular processes such as transcriptional regulation, cell cycle control, apoptosis, and autophagy that are fundamentally involved in tumorigenesis. One further important aspect of HDAC is found in their interaction with non-histone targets. A multitude of non-histone proteins also bind to DNA to affect chromatin structure and exert epigenetic control on gene expression. Thus, HDAC also participate in the formation of protein complexes during the initiation of gene transcription including those of tumor suppressor genes, and the regulation of the acetylation status of specific cell cycle regulatory proteins [13]. The tumor suppressor protein p53 was emerging as the first non-histone target of HDACs and HATs [14]. Acetylation of p53 regulates its binding capacity to specific DNA sequences and thereby its transcriptional factor activity leading to the activation of subordinated target genes [15]. Most importantly, acetylated p53 induces cell apoptosis and basic autophagy by diverse mechanisms which are essential to eliminate transformed cells. An increasing number of studies highlight the role of mutant p53 proteins in tumor development in this regard. The advantage of epigenetic alterations contrary to genetic mutations that are implicated in aberrant tumorigenic gene expression is their reversibility by epigenetic drugs to some extent. Therefore, pharmacological interference in the form of HDAC inhibitors (HDACi) are well suited candidates that are intensively investigated as promising candidates for cancer therapy, in addition to DNA methyltransferase (DNMT) inhibitors [16]. Targeting histone modifying HDACs to restore the expression of tumor suppressor genes has shown clinical benefits as the balance between HAT and HDAC expression is often found disconcerted during tumorigenesis. Although preclinical findings of anti-tumor effects of HDACi seem encouraging, their underlying molecular mechanisms have not been fully clarified to date. They may differ among different HDACi classes and they may be specific for each tumor entity leading to profoundly variable responses by patients in clinical trials. Thus, a deeper knowledge of the mechanisms of action and identification of predictors related to cell death and resistance could pave the way for a more targeted use of HDACi in cancer therapy. This strategy would include the ability to determine who will benefit from a specific HDACi treatment by distinct biomarkers [17].

Recent experimental evidence of our group imply that contingent on the presence or absence of p53 protein in tumor cells [18], HDACi administration could either elicit an apoptotic or autophagic cellular response, respectively. These findings should be integrated into the deliberation of HDACi treatment in clinical trials and help to optimize future therapeutic decisions related to cancer treatment. Nevertheless, confirmation of our experiments with different tumor cell types in vitro and in vivo will be required. Future research will also extend our knowledge of fundamental p53-related function with respect to the modulation of apoptosis, autophagy and their crosstalk. In this review, we discuss the cell death mechanisms elicited by HDACi in cancer treatment, with a special focus on the role of p53 and its involvement in cell death mechanisms.

## 2. Acetylation and Deacetylation of Histones and Non-Histones

Histone deacetylases and HATs form two counteracting, essential, and developmentally safeguarded families of proteins that are master regulators of epigenetic gene expression by modifying the structure of chromatin [19,20]. Even more evolutionary conserved are canonical histones which act as their substrates and belong to the most abundant DNA interacting proteins of eukaryotic cells [21]. As small basic proteins with a high percentage of positively charged lysine and arginine residues, the four core histones H2A, H2B, H3, and H4 share a common structure [22]. A central fold domain is responsible for the octamer formation of the core histones while NH2- and COOH-terminal domains are subject to amino acid residue modification following translation and thereby regulate crucial physiological cellular processes [23]. Histone tail modifications include acetylation, methylation, phosphorylation, sumoylation, and ubiquitination [24,25,26]. The linker histone H1 is required for organizing a higher-order compaction of chromatin, in contrast [27]. By a HAT/HDAC-mediated posttranslational modification of the acetylation status of core histones or non-histone proteins either an open or closed configuration of chromatin is achieved. Consequently, a transcriptionally accessible or suppressive gene structure is generated. In histones this occurs through addition and removal of acetyl groups from the ε-amino lysine residues on histone tails. By specific acetylation at sites of regulatory DNA binding sequences—mostly within promoter regions—active recruitment of transcription factors is granted [28,29]. For instance, acetylation of the histones H3 and H4 usually permits enhanced access and transcription of DNA by forming a tetrameric structure [23]. Beside transcriptional modulation basic cellular processes such as regulation of cell cycle, replication, DNA repair, DNA stress response, apoptosis, autophagy, angiogenesis, and others have been found to be regulated by HDACs [30]. In this sense, a remarkable and increasing number of non-histone substrates have been described for many HDACs and HATs. Non-histone proteins include tumor suppressor proteins (p53, RUNX3), signaling mediators (STAT3, β-catenin, Smad7), steroid receptors (androgen, estrogen, SHP), transcriptional factors and co-regulators (c-Myc, HMG, YY1, EKLF, E2F1, GATA factors, HIF-1α, MyoD, NF-κB, FoxB3), as well as structural (e.g., cell motility proteins), chaperone proteins, and nuclear import proteins (α-tubulin, importin-α, Ku70, HSP90) [31,32,33,34]. Of note, phylogenetic analyses imply that non-histone proteins are the primary intended targets of HDACs as their evolution preceded that of histones [35].

In humans, so far 18 different HDACs are known which have been grouped into four classes according to their structure and function. Histone deacetylases 1, 2, 3, and 8 are structurally related to yeast Rpd3 (reduced potassium dependency-3) protein and belong to class I HDACs. Class II HDACs exhibit homology to yeast Hda1 (histone deacetylases 1) and include HDACs 4, 5, 6, 7, 9 and 10. Histone deacetylases 11 is the single member of class IV. Class I, II, and IV comprises the category of classical HDACs that contain a zinc-binding site while the so-called sirtuins in class III HDACs (SIRT1-7) require NAD+ for their activity. Additionally, different HDAC classification is also based on varying subcellular localization and expression patterns [30,36]. Only class I exhibits ubiquitous expression in the cell nucleus accompanied by the highest enzymatic activity, while other HDAC classes possess a more restricted and distinct tissue-specific expression pattern in nucleus, cytoplasm, or mitochondria.

Different tumor types such as colon, breast, prostate, neuroblastoma, medulloblastoma, and pancreatic carcinoma, however, deviate from the physiological expression pattern of HDACs which renders them primary targets for tumor therapy [13,37,38]. Causes, therefore, have been found in aberrant HDAC recruitment, overexpression, and loss-of-function mutations in HDACs, particularly the loss of acetylation of histone H4 at lysine 16 was found crucial in tumor development [11,39]; and even cell-wide loss of histone acetylation was attested in many tumors. When clarifying the question whether these epigenetic alterations are primary or the consequence of tumorigenesis it was determined that mutations related to the organization of chromatin belong to the most frequent targets in cancer constituting 25–30% of the identified cancer driver mutations [40,41]. Thus, it can be concluded that changes in the epigenetic status are the cause of tumor formation.

## 3. Inhibiting Histone Deacetylases

The aforementioned inappropriate recruitment of HDACs has been shown to lead to transcriptional silencing in cancer cell lines while inhibition of HDACs results in pleiotropic activity including transcriptional reactivation, cell-cycle arrest, and terminal differentiation of transformed cells [42,43,44]. These premises have put HDACi in the center of efforts for identifying new therapeutic anti-cancer treatment with the primary idea that HDACi might reactivate tumorigenesis-associated silenced genes, such as the gene transcribing the cell cycle inhibitor p21 [45]. Although the exact molecular mechanisms of HDACi function still await their elucidation, generally they induce chromatin relaxation and transcriptional de-repression. By restoring or increasing acetylation, re-expression of genes intervening in essential tumor-related functions, such as tumor suppressor proteins, oncoproteins, cell cycle arrest, differentiation, and cell death are enforced [46,47]. Consequently, many clinical evaluations have been performed or are in progress for testing monotherapeutic or combination treatment regimen of different HDACi in hematological and solid malignancies with variable outcomes (www.clinicaltrials.gov) [48,49,50]. To date, four HDACi have been admitted for the treatment of cutaneous T cell lymphoma, multiple myeloma, or peripheral T cell lymphoma (by the US Food and Drug Administration) which are vorinostat (suberoyl hydroxamic acid, SAHA, Zolinza), panobinostat (LBH589), belinostat (PXD-101), and romidepsin (FK228) belonging to different HDACi classes [51,52,53,54,55]. The phylogenic classification of HDACI primarily depends on the chemical nature of their zinc-binding group and comprises the structurally diverse, but small molecule groups, of hydroxamic acids (hydroxamates), cyclic tetrapeptides, benzamides, electrophilic ketones, and aliphatic acids [56]. These groups include natural as well as synthetic compounds. Additionally, most hydoxamates can be categorized as so-called pan- or broad-spectrum inhibitors that inhibit all class I, II and IV HDAC proteins related to their zinc-dependence, while all other groups, with the exception of the HDAC6 isoform-specific tubacin, inhibit all or to some extent members of a specific HDAC class (mostly HDAC class I) [57,58,59]. To date, all licensed HDACi represent pan-inhibitors.

The naturally discovered compound TSA (trichostatin A) and the economically and toxically preferable derivative SAHA are major representatives of the hydroxamates. The bishydroxamate CBHA (m-carboxycinnamic acid bishydroxamate) in this category is the basis for the synthesis of further “second-generation” HDACi such as tubacin, LAQ-824 (dacinostat), LBH-589 (panobinostat), or PXD-101 (belinostat) [58,60,61,62]. The best-known compound of the group of cyclic tetrapeptides is class I-selective FK-228 (romidepsin, FR901228, istodax), a depsipeptide, isolated from *Chromobacterium violaceum*, that is activated upon uptake into cells by glutathione. Trapoxins A and B belonging to this group; however, isolated from the fungus *Helicoma ambiens*, that are too toxic for clinical use [63,64,65]. MS-275 (entinostat) and MGCD0103 (mocetinostat) are representatives for benzamide-based HDACi that also have increased HDAC class I selectivity [66,67,68]. In the category of electrophilic ketones the trifluoromethyl ketone thiazole with significant submicromolar anti-proliferative activity and α-keto amides have been identified [69]. Aliphatic acids in contrast, are less effective HDACi containing the class I- and IIa-specific VPA (valproic acid), PBA (phenylbutyrate) and NaB (sodium butyrate), or AN-9 (pivaloyloxymethyl butyrate) [70,71].

Current research efforts focus on the identification of novel as well as isoform-specific or tissue-selective HDACi with improved efficacy that can be used in mono- or combination therapy [12,57]. HDACi that are specifically developed for HDACs 1–3 are of particular interest as they engage in multiprotein complexes mediating gene transcription [72,73]. This should help to overcome uncovered clinical limitations of tested HDACi related to solid tumor treatment and to reduce off-target effects experienced by patients. Generally, although epigenetic alterations are encountered frequently in solid tumors, only modest efficacy of epigenetic drugs was asserted which might have its origin in abnormal blood supply and vasculature, in intrinsic molecular heterogeneity, and in the associated evolvement of treatment resistance. Clinical studies with HDACi as single agents (e.g., in the treatment of ovarian cancer) have been unsuccessful although previous progress was achieved in preclinical models [74,75]. Phase II trials involving many established HDACi and almost all types of solid tumors, including ovarian cancer, breast cancer, renal cancer, prostate cancer, and head and neck cancer, lacked clinical improvement and were associated with high recurrence of adverse side effects due to their non-selective nature [76,77,78,79,80]. Thus, HDACi were found to induce drug-induced effects extending from slight (diarrhea, anorexia and dehydration) to severe (myelosuppression, thrombocytopenia, and cardiotoxicity) phenotypes [50,81,82]. The molecular explanation for the narrowed impact of HDACi in solid tumors remain puzzling. HDACi-induced compensatory adjustments within tumor cells as well as their cellular microenvironment were suggested to explain the absence of a clinical response. Representative examples, therefore, have been documented by aberrant DNA methylation, HDAC2-truncating mutation, overexpression of HDAC1, and the anti-apoptotic transcription factor nuclear factor NF-κB [83,84,85,86]. Recently, furthermore IκB kinase-dependent expression of proinflammatory chemokines such as IL-8 or CXCL8 have been detected that enhance, in addition to induction of apoptosis, proliferation of tumor cells [87]. Therefore, combined inhibitors for HDACs and IκB kinase should improve the current therapeutic benefits. Some observations also imply that the HDACi applied in clinical studies up to date may lack sufficiently stability to gain access to the solid site of the tumor, and/or that they may not be specific enough for targeting these tumors. Therefore, nanoparticle-supported targeting has been suggested as an additional drug-delivery option, but also here complicating long-term treatment effects are expected [88,89]. Nevertheless, by designing more specific inhibitors, the individual functions of single HDACs are likely to be clarified in more detail, and these continuous efforts may also result in improved efficacy along with reduced adverse effects.

## 4. Molecular Mechanisms of HDAC Inhibition

HDACi possess the capability of restoring aberrant epigenetic alterations such as dysregulated histone acetylation associated with tumor development. Due to the eminent epigenetic regulatory role of the acetylation and deacetylation system, HDACi have far-reaching important effects inducing a host of diverse cellular effects. Although the underlying precise mechanism are still obscure and may vary depending on the specific tumor entity as well as individual treatment regimen, distinct mechanisms can be resolved for many HDACi [82,90,91,92]. One of the major obstacles in this respect is that HDACi not only affects de-repression of HDAC-regulated gene transcription themselves, but also a growing number on non-histone target proteins that are at the beginning of being unraveled. Thus, by regular or exceeding acetylation of histones HDACi trigger, similar to mode of action of HATs, an open state of chromatin and promote gene expression. In addition, for non-histone proteins posttranslational modification of acetylation influences mRNA stability and gene expression, protein binding and stability, protein interactions and enzymatic activity, as well as localization [31]. One of the hallmarks of tumor cells lies in the escape of programmed cell death to support malignant growth. HDACi can revert this process and interfere with cellular growth and stimulate either of the two major morphologically distinctive forms of programmed cell death, namely apoptosis and autophagy [93,94,95,96]. Since this reversible process was found in a variety of tumor cells, HDACi are considered as promising chemotherapeutic agents [97,98]. However, the anti-cancer effects of HDACi go beyond the exclusive re-induction of cell death and possess a broad array of anti-tumor activities. Well-recognized anti-cancer mechanisms of HDACi include, in addition to the promotion of apoptosis, the upregulation of endogenous inhibitors of cell cycle progression, the generation of reactive oxygen species (ROS), the induction of DNA damage, the interference with chaperone protein function, and blocking invasion. Nevertheless, HDACi also exert therapeutic effects that extend beyond cancer and include anti-parasitic, anti-neurodegenerative, anti-rheumatologic, and immunosuppressant activities. In recent years, additional anti-tumor mechanisms of action of these agents have been uncovered. For example, HDACI interfere with multiple DNA repair processes, critical to the maintenance of genomic integrity in the face of diverse genotoxic insults as well as the induction/regulation of autophagy. Nevertheless, considering the array of cellular effects triggered by HDACi, it is probable that several additional mechanisms that still remain to be elucidated contribute to their anticancer activity. Established major molecular activities resulting from HDACi treatment are discussed below.

One very important mechanism of HDACi consists in their ability to re-induce cell cycle arrest and induce cell differentiation in transformed cells [60,99,100,101]. This is due to the fact that HDACi treatment commonly leads to prominent upregulation of p21 (p21cip1/waf1), a cyclin-dependent kinase (CDKN) inhibitor encoded by the *CDKN1A* gene. p53-dependent or -independent expression of p21 in turn causes, by suppressing the formation of dimers from cyclin and CDKN, cell cycle arrest in the G1 or G2 phase of the cell [102,103,104,105]. Acetylation of p53 and its counterplayer HDAC1 thereby seem to regulate promoter binding and transcription of *CDKN1A* oppositely [14,106]. Nevertheless, also the stability of the Runt-related transcription factor 3 (RUNX3) can be modulated by HDACi to influence *CDKN1A* expression and the anti-apoptotic gene *BIM* (Bcl-2-interacting mediator of cell death) [107,108,109,110]. SAHA-induced RUNX3 expression significantly upregulated p21 expression through re-establishment of TGF-β signaling leading to growth arrest in the human biliary cancer cell line Mz-ChA-2 in a further study [111]. Elevated p21 levels not only cause cell cycle arrest but also facilitate the induction of apoptosis [99,112,113,114]. A further direct possibility of HDACi to impede cell cycle progression consists in inhibition of *cyclin D* and *A* gene expression and thereby the activities of CDKN2 and CDKN4 [115]. This inability to pass two cell-cycle checkpoints that are present in normal cells is, according to one model, also representing one of the main explanations for the tumor-selective actions of HDACi [116,117]. In transformed cells, this failure of cell cycle progression results in an early exit from an incomplete mitosis and the subsequent induction of apoptosis [118]. Because the action of HDAC are pivotal to all cells, the effects of HDACi would be considered as cytotoxic for tumor cells as well as normal cells. In contrast to normal cells, however, HDACi treatment should lead to an increased accumulation of DNA damage such as DNA double-strand breaks in sensitive cells such as tumor cells (e.g., by oxidative stress) [119]. In line with this hypothesis, the accumulation of thioredoxin (TXN), an intracellular antioxidant which is a natural scavenger of ROS, was identified in normal, but not transformed, human fibroblasts [120]. Nevertheless, due to the pleiotropic effects of HDACs, transcriptional targets involving hyper-acetylation of chromatin and transcription factors should be considered in the cytotoxic response of HDACi [121].

Treatment of tumor cells with HDACi affects cellular signaling pathways and facilitate cell-cycle arrest, transformed cell differentiation, and/or cell death. Particularly, by modifying acetylation of the non-histone proteins and transcription factors that are involved in cell death signaling (such as NF-κB, p53, and STATs), direct regulation and thereby re-induction of cell death can be achieved [37]. For example, acetylation determines the half-life of the cellular gatekeeper protein p53 by regulating its binding to the mouse double minute 2 homolog (MDM2) E3 ligase, and thereby its proteasomal degradation and transcriptional activity in human non-small cell carcinoma cells H1299 [122]. Also modulation of the WNT pathway via glycogen synthase kinase-3 (GSK-3), that is important for the development of several tumor types, is affected by HDACi [123]. Even proliferation and self-renewal of normal hematopoietic stem cells were found to be regulated by valproic acid–mediated inhibition of GSK-3 and associated activation of the WNT pathway [124].

Many reports highlighting different aspects also implicate HDACi in the interference of DNA damage repair in tumor cells since HDACs are profoundly involved in chromatin-mediated regulation of DNA damage-related proteins [125]. Histone deacetylases 1–3 have been documented to interact with DNA damage sites and modulate deacetylation of histones, which in the case of HDACs 1 and 2 facilitate non-homologous end-joining presumably during double-strand break repair [126,127]; nevertheless, also the expression of DNA damage-related response proteins (ATR, ATM, BRCA1, FUS) is regulated by class I HDACs [128]. But also, class II HDACs and sirtuins are involved in the repair of DNA damage. Histone deacetylase 4 is localized together with 53BP1, a homologous recombination repair protein, at DNA damage-induced foci, and deletion or inhibition of HDAC9 and 10 directly impairs the process of homologous recombination [129,130]. Inhibition of HDAC6 even causes cell death by interfering with MSH2-regulated DNA mismatch repair capability of the cell [131]. SIRT1 is involved in many processes of DNA damage response that include signal transduction, sensing and repair of DNA damage, as well as apoptosis [132]. This can be achieved by de-acetylating and binding to a variety of DNA-binding and -repair proteins (Ku70, NBS1, APE1, XPA, PARP-1, TopBP1, KAP1) that are involved in non-homologous end joining, the repair of double strand break by homologous recombination, and base or nucleotide excision repair [133]. DNA damage-induced apoptosis can be induced by p53-mediated target gene expression if SIRT1 is phosphorylated which prevents the SIRT1-mediated deacetylation activity of p53 [134]. SIRT6 has been found to be important for homologous recombination during DNA double strand break repair and base excision repair [135,136]. As a cause for the tumor cell-specific activity of HDACi, SAHA treatment was demonstrated to continuously increase the expression of phosphorylated H2AX, a marker of DNA double strand breaks in transformed but not in normal cells [119].

A further detrimental effect of HDACi concerns the inhibition of angiogenesis [137,138]. Early tumor angiogenesis can be blocked by HDACi-mediated hyperacetylation of HIF-1α and subsequent degradation of this hypoxia-induced transcription factor [139]. Moreover, HDACi disable tumor angiogenesis by attenuation of vascular endothelial growth factor receptor (VEGFR) expression [138]. Since angiogenesis is a crucial process, both mechanisms certainly enhance the tumor-suppressive effects of HDACi, and therefore, tumorigenesis or metastasis at later stages. Additionally, anti-angiogenic effects of HDACi have been associated with altered expression of many pro- and anti-angiogenic genes [140,141].

Also, immunomodulatory responses that are involved in many cellular processes have been delineated as HDACi-stimulated effects [34,142]. Via multiple mechanisms, HDACi increase the immunogenicity of tumor cells. Thus, exposure of tumor cells to HDACi helps in upregulation and translocation of antigen presenting and co-stimulating molecules on their surface such as MHC class I and II molecules, as well as natural killer (NK)-cell-activating ligands, or calreticulin [143,144]. HDACi pre-exposed malignant cells are therefore more susceptible for T cell mediated lysis and dendritic cells or have been used to generate effective anticancer vaccines [145,146]. Also, number and function of different lymphocytic cells, such as T cells and NK cells, were increased by class I and II HDACi [147].

Additionally, tumor survival is hampered intracellularly by inhibition of stress response pathways in the endoplasmic reticulum which effects proper elimination of misfolded proteins [101,112]. Thereby, the stability and expression of oncoproteins can be regulated. By LAQ824-induced inhibition of HDAC6 and consequent acetylation of its substrate HSP90, disruption of the chaperone function related to pro-growth and pro-survival oncoproteins (e.g., BCR-ABL, mutant FLT-3, c-RAF, AKT, c-KIT, Her-2, BRAF) was documented resulting in their inappropriate degradation in human leukemia cells [46]. Thus, a combination of HDACi with either HSP90 inhibitors, tyrosine kinase inhibitors, or proteasome inhibitors might be additionally favorable. In this regard, also selective inhibition of HDAC6 led to inappropriate degradation of unfolded, and misfolded ubiquitinated proteins that are degraded not only by proteasomes but also by HDAC6-dependent aggresomes [148]; using a combination of bortezomib and tubacin, caspase-dependent apoptosis caused by the accumulation of ubiquitylated proteins in multiple myeloma cells was induced. Interestingly, unlike other HDAC, HDAC6 was identified to mainly catalyze epigenetic regulation of cytoplasmic proteins which beside HSP90 also includes α-tubulin and cortactin [117]. Increased acetylation of α-tubulin was found to facilitate dynesin and kinesin binding of microtubules and thereby enhance routing into early endosomes [149]. Thus, HDAC6 silencing by specific shRNA was very recently also demonstrated to impair leukemia outgrowth in xenografted mice associated with increased Notch3 accumulation in lysosomes and elevated apoptosis of T-cell acute lymphoblastic leukemia cells [150].

Overall, genome-wide epigenomic alterations of chromatin originating in HDACi treatment is exceptionally well accepted by a large number of eukaryotic cells. As many consequences of HDACi intervention are found downstream of transcription factors triggering a complex network of cellular responses they are hard to identify. Re-induction of less than 2% of expressed genes were shown to be influenced by genome-wide hyperacetylation following HDACi treatment including histone and non-histone proteins that connect transcriptional and non-transcriptional effects [125,151]. It has been demonstrated that in human lymphoblastoid cells as an early response to HDACi treatment by valproic acid and SAHA, a strong increase in H3K27me3 at transcription start sites has been detected that proved to be independent of transcriptional requirements. It was assumed that this change provides an adaptive measurement to promote cell survival by minimizing protein hyperacetylation, slowing growth, and re-balancing patterns [152]. Following this initial modulatory survival step, HDACi can exert further downstream its other forms of epigenetic control. Thus, the inhibition of deacetylation may affect the genome at multiple levels including DNA and histone methylation, or even microRNA expression in a spectrum of different tumors [153,154]. Nevertheless, to exploit the entire potential of HDACi for clinical use, more specific information with regard to HDACi-mediated signaling involved in epigenetic intervention will be required.

## 5. HDAC Inhibitor-Induced Apoptosis

Induction of apoptosis following HDACi-mediated hyperacetylation is well documented and the prevailing form of cell death [155]. In various tumor cell lines, treatment with HDACi most frequently induces apoptosis by sequential activation of a series of cysteine-dependent aspartate-directed proteases, termed caspases [156,157]. Either one or both pathways of this programmed form of type-I cell death are engaged by HDACs depending on the type of cancer, the intrinsic (mitochondrial) pathway and the extrinsic (death-receptor) pathway [156,158]. As a common motif HDACi primarily seem to influence the balance between pro- and anti-apoptotic proteins by interfering with their expression [97,159]. Both modes of apoptosis, the extrinsic as well as intrinsic pathway, finally activate executioner caspases inevitably leading to degradation and death of the cell. Thereby, HDACi selectively induce apoptosis in transformed cells, while skipping normal cells reflecting one of their most encouraging characteristics as described in Section 4 [97].

The intrinsic pathway leading to mitochondrial membrane disruption, release of cytochrome C, apoptosome formation, and caspase-9 activation can be induced by diverse chemical agents and the upregulation of pro-apoptotic proteins of the Bcl-2 family (e.g., BAX and BAK) including the BH3-only proteins (BIM, BMF, BAD, BID) that control stability of the mitochondrial membrane [102,118,160,161,162]. While BAX and BAK are indispensable for apoptosis, BH3-only proteins include modifiers, i.e., activator proteins (e.g., BIM) as well as sensitizer or derepressor proteins (e.g., NOXA, BIK) [163]. In the case of Bid, even SAHA-mediated cleavage of the protein was reported [118]. Primarily, post-translational modifications of the transcription factor p53 preventing its degradation is a HDACi-governed mechanism that promotes cell cycle arrest and the expression of pro-apoptotic genes [164]. This interferes with the ability of p53 to induce apoptosis via transcriptional transactivation of many pro-apoptotic genes, particularly *BAX*, *PUMA* and *NOXA*. Nevertheless, HDACi can stimulate apoptosis independently of p53 and similarly to p53, hyperacetylation of RUNX3 by HDACi increases stability and transcriptional activity, thereby leading to cell cycle arrest and apoptosis by transcriptional upregulation of p21 and Bim in tumor cells [108,165,166]. The expression of anti-apoptotic genes, such as BCL-2, *BCL-XL*, *XIAP*, *MCL-1*, or *SURVIVIN* in contrast will be downregulated by HDACi which in the case of *BCLcl-2* can be mediated by extracellular-related signal kinase (ERK) activation [102,103,167,168]. Accordingly, overexpression of *BCL-2* and *BCL-XL* was found to inhibit HDACi-mediated apoptosis [118,156].

In the extrinsic pathway, activation of caspase-8 and recruitment of the FADD adapter protein depends on binding of death receptors DR4 and DR5 by corresponding ligands such as TNF-related apoptosis-inducing ligand (TRAIL) [169,170]. HDACi-induced reactivated expression of TNF-superfamily members such as TRAIL, DR5, DR4, FAS, FAS-L, and TNF-a restores sensitivity, and therefore, helps to overcome crucial steps that are blocking extrinsic apoptosis [171,172,173,174,175,176,177,178]. But also butyrate-dependent reduction of the expression of the anti-apoptotic protein c-Flip was reported as a mechanism supporting the extrinsic apoptotic pathway [179]. Death-receptor mediated cell death facilitated by HDACi treatment might also explain favored distinct induction of apoptosis in malignant cells and gives the rationale for clinical combination studies of HDACi with TRAIL or agonistic antibodies.

Additionally, HDACi are able to promote ROS-dependent apoptosis and the associated induction of DNA damage by upregulation of pro-apoptotic proteins which promote the intrinsic pathway [101,118,120,180,181]. The precise mechanism leading to ROS accumulation is unclear but might be related to the fact that upon HDACi treatment the ROS scavenger TRX is present in normal fibroblast cells, and therefore, able to compensate oxidative stress which is not the case in malignant cells [120]. Thus, elevated expression of the TRX binding protein-2 (TBP-2) that possesses inhibitory activity could be demonstrated by combinatorial treatment of prostate, bladder, and breast tumor cells with SAHA and MS-275/entinostat [101]. Overall, beside the activation of death receptor pathways the ability of transformed cells to escape HDACi-induced oxidative injury might be a further reason for the selective cytotoxicity of HDACi for neoplastic cells.

Furthermore, HDACi-induced release of the cytoplasmic Ku70, a DNA repair protein that interacts with Bax in an acetylation-sensitive manner has been demonstrated to trigger mitochondria-mediated apoptosis in neuroblastoma cells [182]. This could involve also acetylation of p53 whose transcription-independent functions are required for BAX activation, ROS generation, and apoptosis in response to HDACi SAHA and LAQ824. As underlying mechanism, interaction between p53 and Ku70 was detected that could disrupt the Ku70-BAX complex and thus enhance apoptosis. This could be demonstrated by deleting endogenous mutant p53 in cancer cells which significantly reduced HDACi-induced cytotoxicity, whereas expression of transactivation-deficient p53 variants sensitized *p53*-null cells to HDACi mediated BAX-dependent apoptosis.

In recent years, HDACi have been implicated in the epigenetic control of non-coding RNA (ncRNA) expression that evolved as crucial players in cancer biology [183]. The human genome encodes in addition to a small proportion of genes encoding for proteins also a large number of ncRNAs that include microRNAs (miRNA/miRs), small interfering RNAs (siRNAs), PIWI-associated RNAs (piRNAs), and long non-coding RNAs (lncRNAs) [184]. These are processed to regulatory RNAs that interfere with gene expression by binding to the 3′-UTRs of target mRNAs which results in gene silencing via degradation of the target mRNAs or translation repression [185,186]. Many miRNAs were found to be involved in the regulation of cellular proliferation and differentiation, and also have been implicated in multiple tumor-related processes such as migration, invasion, epithelial to mesenchymal transition, and metastasis, particular aberrant miRNA expression patterns might be identified not only as molecular key players, but also promising epigenetic markers for cancer diagnosis [187,188,189,190,191]. Even lncRNAs which due to their size beyond 200 nucleotides are capable of forming secondary and higher-order structures, have been attributed with important tumorigenesis-associated functions as for example lncRNA MEG3 modulates the proper tumor suppressor function of the p53 protein [192,193]. Of particular interest related to epigenetic modifier-mediated anti-tumorigenic mechanisms are miRNAs that have been described to regulate epigenetic regulatory genes such as *DNMTs* and *HDACs* [194,195]. Both, the abundance of miRNAs as well as lncRNAs, which have been both implicated in tumor development, have been altered by HDACi treatment [196]. Thus, it has been reported that the re-establishment of ncRNA expression also contributes to the apoptotic response incurred by HDACi. For example in thyroid cancer cells, induction of apoptotic cell death could be enforced by TSA and SAHA-induced overexpression of miR-129-5p [197]. Induction of apoptosis by downregulation of the anti-apoptotic genes *BCL-2* and *BCL-XL* was furthermore achieved in B-cell lymphomas and non-malignant B-cells by MYC-mediated transcriptional repression of the miR-15 and let-7 miRNA families following HDACi treatment [198]. Additionally, sodium butyrate and panobinostat-stimulated expression of *miR-31-*activated cellular senescence through BIM1-mediated repression in breast cancer cells and fibroblasts [199]. In pancreatic cancer, *miR-34a* was reported to be downregulated and act as a tumor suppressor [17].Upon SAHA treatment, even a crosstalk between acetylation and *miR-34a* was revealed, as the re-induction of *miR-34* not only induced caspase-3/7-dependent apoptosis, but also activated acetylation of p53 and thereby transcriptional activation of its target genes *CDKN1A* and *PUMA*. Indications for the involvement of lncRNAs that act on chromatin complexes or RNA binding proteins for influencing gene expression come from their detected aberrant expression in TSA-treated hepatocellular cancer cell lines [200]. By HDAC1-mediated acetylation of DGCR8 (DiGeorge syndrome chromosomal region 8), a protein required for microprocessor complex formation (together with Drosha) that supports miRNA cleavage and stem loop formation, decreased miRNA expression on an even broader scale [201]. Vice versa, several HDACs can also be targeted by miRNA, for example, repression of HDAC1 by *miR-449a* in prostate cancer cells, thereby phenocopying the effects of HDACi [202]. Interestingly, miRNAs could also be subject to epigenetic silencing mechanisms themselves, as it has been demonstrated that putative tumor suppressor miRNAs (such as *miR-127*, *miR-124a*, and *miR-34b/c*) are methylated in tumor cells [203]. This adds a new level of complexity in the tumorigenic regulation of ncRNAs and will warrant many questions for this area of research in coming years.

Although epigenetic drugs can be used as monotherapy, their clinical efficacy can be optimized by combination therapies. Thus, combinatorial treatments of several HDACi (e.g., SAHA, depsipeptide, MS-275, and TSA) with many other synergistic agents have been established. These include well-known chemotherapeutic drugs with different cytostatic tumor-killing effects such as gemcitabine, paclitaxel, cisplatin, etoposide, doxorubicin, and epirubicin [204,205,206,207,208]. A further important category of co-effectors are found in epigenetic co-modifiers such as DNA methyltransferase (DNMT) inhibitors which are members of nucleoside analogue family, for example the demethylating agents azacitidine (5-azacytidine) and decitabine (5-aza-2′-deoxycytidine) or the less toxic variant zebularine (derived from 5-azacytidine), as well as transcriptional modulators such as retinoic acid which allow reactivation of epigenetically silenced genes by induction of global hypomethylation. [83,209,210,211,212]. As determined by experiments of our own and other groups, re-expression of key apoptotic and presumably also autophagic genes is facilitated by this treatment allowing transformed cells to continue their exit by programmed cell death. To overcome limiting steps in cell death signaling by directly stimulating the apoptotic program, HDACi therapy also benefited from addition of anti-TRAIL receptor agonists, such as TRAIL or agonistic antibodies [155,173,210,213]. This combination was considered to be very tumor-selective, but also relatively harmless for non-malignant cells [214]. The underlying mechanism seems to lie in the higher sensitivity of malignant cells towards TRAIL-induced apoptosis while the cell cycle arrests [160,174,215]. As p21-mediated cell cycle arrest might also hamper the apoptosis program in return, currently there is a search for synergistically applicable p21 inhibitors (e.g., flavopiridol and sorafenib) [216,217]. Furthermore, combinatorial effects of class I-specific HDACi affecting HDAC1 or HDAC2 activity and TRAIL (such as SAHA, MS-275, and depsipeptide) help to overcome TRAIL resistance by higher expression of cell death receptors and the associated formation of signal complexes [171,174,178,210]. As a further generally common mechanism of synergistic HDACi treatment, the threshold of apoptosis induction seems to get decreased by direct or indirect interference with the expression of pro- or anti-apoptotic molecules, respectively [121,155,218]. This explanation was based on the lowering of anti-apoptotic protein levels (XIAP, SURVIVIN, and BCL-2) on the one hand and on the elevation of pro-apoptotic protein levels (bim, bmf, and bid) on the other hand [101,118,160,161,168,215,219,220]. In future, the combination of HDACi with other novel developed drugs such siRNA might additionally enhance their clinical utility for many current therapies [221].

## 6. HDAC Inhibitor-Induced Autophagy

**Figure 1:**
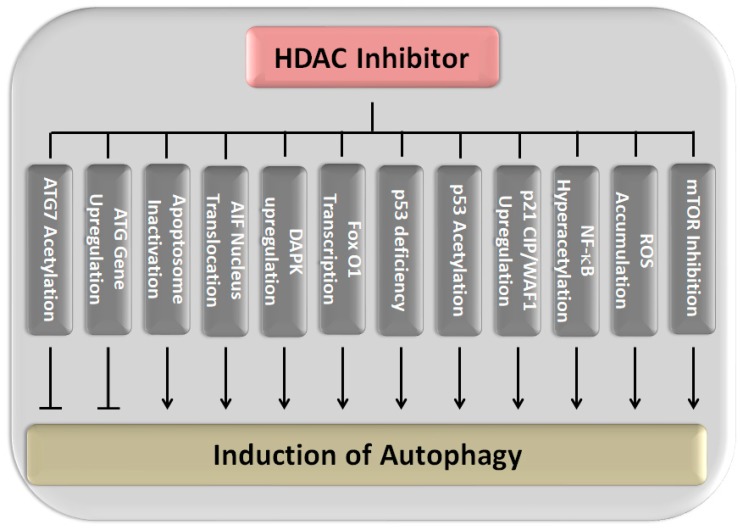
Known signaling pathways involved in histone deacetylase inhibitor-elicited activation or suppression of autophagy. In most cases mTOR inhibition, ROS accumulation, NF-κB hyperacetylation, p21 upregulation, or the involvement of p53 signaling is observed.

Autophagy (also called cell-death type II) as an anti-tumor response has been added to the list of HDACi-mediated effects only very late [222]. Autophagy is a complex conserved and genetically controlled process resulting in the degradation of cytoplasmic constituents within specific lysosomes so called autophagosomes [223,224]; due to its paradoxical relationship with apoptosis, autophagy has attracted much attention. In wild-type cells, autophagy occurs at a basal level and represents a tumor-suppressor mechanism, whose disruption causes oxidative stress, DNA damage, and genomic instability among other effects that predispose for tumor development. Under stressful physiological conditions including starvation, hypoxia, growth factor withdrawal, and senescence, as well as pathological conditions such as tumor, autophagy can be stimulated above basal levels. As the role of autophagy in tumor therapy could be either cytoprotective or cytotoxic, the benefit of HDACi-induced autophagy is highly debated. Specific conditions promoting cell survival or cell death seem to depend on the cell type and genetic predisposition of the tumor as well as the duration and dose of the HDACi, [225,226,227,228]. Thus, on the one hand, autophagy was considered indispensable in the elimination of SAHA-treated apoptosis-resistant uterine sarcoma cells or SAHA and OSU-HDAC42-treated hepatocellular carcinoma cells [18,229]. On the other hand, it was demonstrated that inhibition of autophagy by RNAi promoted SAHA-induced apoptosis in glioblastoma cells [230]. As an underlying mechanism, a variety of signaling pathways that initiate the activation or suppression of autophagy have been unveiled for HDACi-mediated autophagy as reviewed and listed in detail previously (see Figure 1) [231]. The primary motif hereby seems to be found in the interference with the acetylation of many autophagy-related proteins and particularly autophagy-related gene products (ATGs) such as ULK1, ATG3, or ATG7 that is driven by the balance between HAT and their corresponding HDAC [82,232].

Most studies reporting about HDAC-induced autophagy observed inhibition of the nutrient-sensing kinase mammalian target of rapamycin (mTOR) [18,222,229,233,234,235,236,237,238]. Additional transcriptional upregulation of autophagic key regulators such as LC3, BECLIN-1, and ATG proteins was found in several cases. mTOR is a major suppressive regulator of autophagy that phosphorylates and thereby inactivates the ULK1 complex, an upstream component of the autophagic signaling machinery. mTOR inactivation by SAHA, for example, restores the function of the ULK1 complex and thereby induces autophagy [229,233,234,235]. In several reports, concurrent induction of autophagy as well as apoptosis was determined. For example, by the treatment of HeLa cells with SAHA and sodium butyrate, apoptosis as well as autophagic cell death independent of caspase activation was demonstrated by ultrastructural changes in HeLa cells [222]. Caspase independency in this case was proven by ultrastructural changes and overexpression of BCL-XL inhibited cytochrome C release and caspase activation, but not HDACi-mediated autophagy. Inactivation of mTOR activity as well as the formation of autophagosomes in a BECLIN-1- and ATG7-dependent manner could be determined in a subsequent report [237]. The decisive role of mTOR in the regulation of SAHA-induced autophagy could be confirmed later by studies of our own group on endometrial sarcoma cells and by Gammoh et al. [18,233,236]. The crucial function of the ULK1 complex in this pathway was further stressed by the finding that SAHA is not able to promote autophagy in ULK1-deficient cells. Acetylation of ULK-1 initiated by activation of the histone acetyltransferase TIP60 via glycogen synthase kinase-3 (GSK3) also represents a physiological mechanism found during starvation-induced autophagy [239]. In a further example supporting interacting apoptosis and autophagy, mocetinostat/MGCD0103 treatment led to diminished autophagy by activation of the PI3K-AKT-mTOR pathway in primary chronic leukocytic leukemia cells, as determined by the autophagic markers MAP1LC3-II and SQSTM1/p62 [238]. This suppression of autophagy was caused by caspase- and calpain-1-mediated cleavage of ATG proteins, but presumably also by transcriptional downregulation of autophagic key regulators. When mocetinostat treatment induced autophagy in other tumor cell lines such as MCF-7, levels of MAP1LC3-II and ATG5 to ATG12 proteins were found increased there. These findings represent a perfect example for a cell-line specific mode of action, further indicating that basal autophagic activity acts as a pro-survival mechanism under normal conditions, whereas its disruption potentiates cell death.

A second important mechanism leading to HDACi-mediated autophagy, is the generation of ROS accumulation which can also be found in combination with mTOR attenuation. Thus, SAHA treatment has been found beside mTOR attenuation to enhance massive intracellular ROS generation by disrupting mitochondrial respiration and energy metabolism and to induce autophagy. In the case of romidepsin, the proteasome inhibitor bortezomib could furthermore enhance autophagic cytotoxicity [240]. Transcriptional upregulation of the lysosomal protease cathepsin D or suppression of its substrate, TRX, as well as activation of MAPK family members such as ERK1/2 and JNK has been additionally determined in several tumor cells demonstrating HDACi-dependent generation of ROS [234,241]. The exact targets of HDAC activity causing ROS formation are unclear thus far, but could be found in posttranslational modified regulatory proteins, such as TRX. Proteomic analysis of SAHA-induced Jurkat T-leukemia cells provided evidence for the upregulation of enzymes related to energy metabolism, anti-oxidative stress, and cellular redox control [234,242]. Also, here, HDACi treatment makes use of the fact that tumor cells have reduced ability to handle oxidative injury as already lined for ROS-induced apoptosis.

Induction of NF-κB target genes by NF-κB RELA/p65 hyperacetylation elicited by SAHA and MS-275 treatment of PC3 cells was found to activate autophagy and suppress the innate immune system in vesicular stomatitis virus oncolysis without detailing the underlying process [243]. Downregulation of pERK/NF-κB signaling and upregulation of p21 was concluded as the cause of HDACi-induced autophagy from two other studies. In PC-3M and HL-60 cells, H40, a novel sulfur-containing hydroxamate, and SAHA induced cell differentiation, cell cycle arrest, and autophagy by hyperacetylation of histone H3 and p21CIP/WAF1 expression. [244]. Also in PC3 prostate cancer cells the novel HDACi MRJF4 activated autophagy [245].

HDACi-dependent autophagic induction involving p53 acetylation and p53-deficiency defined on the basis of our findings will be discussed in detail below.

Single reports also implicate nuclear translocation of the apoptosis inducing factor (AIF), apoptosome inactivation, transcriptional activity by FoxO1, and the upregulation of death-associated protein kinase (DAPK) expression as regulatory mechanisms in HDACi-induced autophagy [18,243,246,247,248,249]. Nuclear translocation of AIF was found as the cause of apoptosis, necrosis, or autophagy induced in malignant rhabdoid tumor cells that were treated with class I and II HDACi FK228/depsipeptide [246]. This could be proven by disruption of autophagy through targeted deletion of AIF that translocates into the nucleus where it causes caspase-independent cell death. Apoptosome inactivation by Apaf-1 or caspase-9 deletion, suppressing the late stages of apoptosis, led to induction of autophagy as indicated by morphologic and biochemical characteristics upon treating Eu-lymphomas with LAQ824/dacinostat and LBH589/panobinostat [249]. Activation of the transcription factor FOXO1 by SAHA and TSA was revealed as a further cause of HDACi-induced autophagy [249]. Upregulation of FoxO1 expression resulted in sestrin 3 (SESN3)-mediated mTOR-suppression and upregulation of ATG expression in HepG2 and HCT116 cells thereby promoting autophagy. DAPK is a calcium/calmodulin modulated cytoskeleton-associated enzyme associating with different MAPKs such as ERK in response to inflammatory apoptotic stimuli [248]. Activation by LBH589/panobinostat-induced dephosphorylation of serin308 was elucidated as the cause for DAPK protein interactions promoting autophagy in HCT116 colon cancer cells rather than its catalytic function [176].

In two reports HDACi-mediated suppression of autophagy was also noted. Thus, repression of the regulatory autophagic protein ATG7 and interacting proteins on transcriptional as well as posttranslational level, was held responsible for concurrent promotion of apoptosis and decreasing the autophagic flux in myeloid leukemic cells. Acetylation levels of these proteins were modified following single treatment with valproic acid, SAHA, TSA, panobinostat, or JQ2, a specific HDAC1 and -2 inhibitor. A knockdown of ATG7 could therefore recapitulate these effects [250]. In addition, Tenovin-6, an inhibitor of sirtuins (i.e., NAD^+^-dependent class III HDAC), was documented to inhibit the late stages of autophagy in chronic lymphocytic leukemia (CLL) cells independent of p53 activation [251]. This was evident by upregulation of autophagy-lysosomal pathway genes and increased expression of the autophagic markers LC3-II and p62 as well as altered cellular ultrastructure.

Also, several HDACs themselves have been reported to induce autophagy by different mechanisms providing a novel basis for targeting the autophagic process. In contrast to HDACi, which have been primarily established as stimulatory modulators of autophagy by deacetylating ATG proteins, HDAC have been associated with inhibitory effects on autophagy. HDACi-triggered autophagy has been linked to HDAC1 either by the HDACi FK228 in HeLa cells or to HDAC1 and 2 by TSA in phenylephrine-induced autophagy of cultured cardiomyocytes involving the autophagic effector molecules ATG5 or Beclin-1 [252,253]. HDAC6 was identified as a microtubule-associated deacetylase that induces autophagy following an impaired ubiquitin-proteasome-system. HDAC6 which can be inhibited by the HDACi tubacin, binds to polyubiquitinated proteins and is essential for autophagosome-lysosome fusion [158]. Apicidin-mediated suppression of HDAC7 in salivary mucoepidermoid carcinoma (MEC) cells resulted in cell cycle arrest, induction of apoptosis and autophagy, as well as reduced ERK activation [254]. Furthermore, HDAC10 was reported to mediate autophagic cell survival in neuroblastoma cells which could be disrupted using the class IIb inhibitors bufexamac and tubastatin [255]. For the NAD^+^-dependent deacetylase Sirt1, an important regulatory role for autophagy was figured out as it associates and directly deacetylates several autophagic components such as ATG5, ATG7, and ATG8 [256]. Sirt2, in contrast, interferes with the acetylation of the transcription factor FoxO1, thereby preventing its interaction with ATG7 that stimulates autophagy. Overexpression of Sirt6 was moreover documented to promote autophagic induction via the IGF-Akt signaling axis in human bronchial epithelial cells or ROS production under oxidative stress in neuronal cells thereby attenuating mTOR [257,258].

## 7. HDACi-Mediated Acetylation of the Non-Histone Protein p53

HDACi potentiate their anticancer-effects by preventing deacetylation of non-histone proteins by HDACs that control many crucial cellular functions with respect to growth, differentiation, migration, senescence, and death [90]. Acetylation of the tumor suppressor protein p53, a master regulator of cell integrity and homeostasis, with a fundamental role in the prevention of tumor development, was the first identified non-histone target in this regard [14,259]. Integration of a multitude of extra- and intracellular stress signals such as DNA damage, oncogene activation, DNA methylation alterations, genotoxicity, hypoxia, and oxidative stress that can be sensed by p53. These signals regulate the “gatekeeper of the cell” by posttranslational modifications of the protein which are not fully elucidated yet, although it is one of the most widely studied molecules. Beside acetylation, these also include phosphorylation, ubiquitination, neddylation, and sumoylation that determine, among other properties, nuclear export and proteasomal degradation of p53 as it exerts only a short half-life under normal physiological conditions [260]. Acetylated residues attached by several HATs can be found for p53 at distinct sites which increases its stability and promotes sequence-specific DNA binding leading to increased transcriptional activity at target genes [14,15]. Interference by deacetylation of HDACi enable on the one hand the accessibility of p53 to its target genes and on the other hand also could affect co-activator recruitment, nuclear export, or proteasomal degradation of p53 itself [261,262,263,264]. For example, a complete loss of p53-dependent p21 transcription could be demonstrated by mutating C-terminal acetylation sites of p53 [265]. Proteasomal degradation of wild-type as well as mutant p53 is predominantly regulated by the activity of the ubiquitin ligase MDM2 whose activity is controlled by acetylation itself [266]. Nevertheless, as mutant p53 exceeds the levels of wild-type protein by far it is able to escape MDM2-mediated degradation [267].

Impairment of p53 wild-type function, particularly by missense mutations provoking a single amino acid change in the DNA-binding domain which results in loss of sequence-specific DNA binding to the canonical wild-type p53 binding sites of target genes, represent the most frequent genetic alterations found in human tumors [267,268]. Very often these heterozygous mutant variants of p53 are stabilized and accumulated in the cell, thereby applying a dominant-negative effect over the remaining wild-type allele, e.g., by preventing its binding to the promoter, or by even acquiring even pro-oncogenic functions [269,270,271]. Such gain-of-function alleles are characterized by hyperstability due to overexpressed chaperone or co-chaperone proteins (such as HSP90, BAG family proteins) or MDM2 short isoforms and signify less chemotherapeutic success for patients [272,273,274]. In this context, a remarkable finding investigating the cytotoxic role of HDACi described a destabilizing effect on mutant p53 protein by polyubiquitination and proteasomal degradation [275,276]. Therefore, it was concluded that only mutant but not wild-type or p53-null mutants render cells sensitive to HDACi as exemplified by TSA, FR901228, or SAHA treatment. Furthermore, HDACi administration in the presence of mutant p53 led to transcriptional reactivation of p53-dependent transcription [276]. Either by re-establishing or copying the trans-activating functions of p53, significant upregulation of p21 and MDM-2 expression could be documented that implicated the degradation of mutant p53 [277]. However, it could be possible that also autophagy is involved in HDACi-mediated reduction of mutant p53 expression [278,279]. SAHA-induced inhibition of HDAC6 was reported as an additional mechanism leading to the release of mutant p53 from the chaperone HSP90 and facilitating its degradation by MDM2 and CHIP ligases [280]. In contrast, by another study elevated mutant mRNA and protein expression levels of p53 could be elicited by either ectopic expression of HDAC8 or addition of SAHA or sodium butyrate/NaB in tumor cells which was mediated by the HoxA5 transcription factor [281].

## 8. Role of the Mutational Status of p53 for HDACi-Induced Cell Death

As previously noted, induction of caspase-induced apoptosis, frequently in combination with other HDACi-mediated effects such as cell cycle arrest and ROS generation, was resolved as the most common type of HDACi-mediated cell death [155,156,158]. But in addition, experimental evidence amounts that in response to several HDACi, such as SAHA, alternatively or additionally autophagic cell death can be stimulated in tumor cells and therefore offers high potential for future therapy and is intensively investigated [18,222,229,233,236,237,248,282,283,284]. In contrast to apoptosis or necrosis, autophagy takes over a pro-survival or a pro-death role if activated in tumor cells [227,285]. This might be of decisive advantage for frequently present apoptosis resistance due to a variety of defects in caspase-mediated pathways in tumor cells where autophagy rather pursues a tumor suppressive, and thus, cytoprotective function [227,247,250,286,287,288]. Beside restraining tumor necrosis and inflammation, this mode can furthermore assist the cells to deal with metabolic stress and cytotoxicity during chemotherapy. Under certain but unknown conditions, however, autophagy may promote cell death by accelerating the autophagic pathway in the tumor cell using unclear mechanisms [289]. Another scenario could be that expedited autophagy might also sustain the tumor by serving its higher metabolic turnover. In this context, disruption of the autophagic program will facilitate tumor survival. Moreover, enhancing autophagy provides also a promising target to avoid acquired HDACi-resistance.

Despite these insights, in recent years clinical combination strategies have intensively favored HDACi treatment combined with agents that disrupt autophagy for cancer therapy; using this treatment option the pro-apoptotic effects of HDACi should be enhanced by inhibition of autophagy. This strategy is supported for example by an early study discovering that autophagy blockade significantly augmented SAHA-mediated apoptosis in chronic myelogenous leukemia cell lines and primary cells [241]. Inhibition of autophagy by 3-methyladenine following sirtinol treatment also facilitated increased apoptotic cell death in MCF-7 cells [290]. Similarly, another report demonstrated broad anti-cancer activity of a novel hydroxamic acid derivate CTS203 against MCF-7 breast cancer cells by triggering apoptosis and autophagy. Further addition of the autophagic inhibitor 3-MA supported the cleavage of Beclin-1, and conclusively enhanced apoptotic cell death via a caspase-9-dependent pathway. In contrast, butyrate and SAHA treatment resulted in the activation of apoptosis and autophagy in HeLa cells [291]. Inactivation of caspase-dependent cell death type-I by Apaf-1 deletion, overexpression of Bcl-XL, or pharmacological inhibition of caspase activity did not prevent HDACi-induced cell death, however, supporting a prominent role for autophagic cell death. In the long run, combination strategies using modulators of autophagy and HDACi for the treatment of malignancies will be of decisive advantage as de novo or acquired resistance to HDACi therapy is inevitable [17,292,293]. Consistently, inhibition of autophagy by knockdown of Beclin-1 or Lamp-2 restored sensitivity to HDACi treatment in developed SAHA-resistant clones of the hematological cancer cell line U937 [294].

A further strategy particularly in apoptosis-resistant tumor cells, that are often deficient for the tumor suppressor protein p53, could lie in enhancement of HDACi-activated autophagy. With respect to this idea, a study of our own group previously uncovered a p53 mutant in endometrial stroma sarcoma cells that denotes a master regulatory role for p53 in driving HDACi-mediated autophagic and apoptotic cell death [18]. Consistent with a tumor suppressive role, we observed that HDACi treatment mediated autophagic, caspase-independent cytotoxicity in uterine sarcoma sarcoma cells [236]. Promoted by SAHA treatment, we previously demonstrated predominant dose-dependent activation of autophagy in ESS-1 cells, but prevalent induction of apoptosis in MES-SA cells [210,236,295]. Cell death resulting in elimination of 80% ESS-1 cells and 48% MES-SA cells after 24 h of SAHA treatment, respectively, was accompanied by upregulation of p21 and cell cycle arrest at the G1/S transition phase. Upon closer molecular investigation the attenuation of mTOR expression was detected in ESS-1 cells triggering the autophagic pathway [236]. Nevertheless, in a follow-up study, we tackled the question which upstream signaling pathways connect mTOR with SAHA-induced autophagy [18]. By screening key regulators of apoptosis and autophagy upstream of mTOR, the lack of p53 protein and decreased levels of PUMA (p53 upregulated modulator of apoptosis) expression could be uncovered. Lack of p53 expression was presumed to be caused by an identified novel R213X nonsense mutation located in the transactivating domain of p53 in ESS-1 cells that obviously leads to degradation of the transcript. By rescuing p53-deficiency in ESS-1 cells, re-induction of the apoptotic pathway could be initiated which was supported by increased PUMA and caspase-9 expression, activation of caspases-3 and -7, and PARP-1 cleavage. Concurrently, mTOR levels were raised again that re-established basic autophagy as confirmed by LC3 and MDC staining. Thus, due to the functional status of the tumor suppressor protein p53 in the cell a switch between apoptosis and autophagy occurred. This HDACi-dependent p53-mediated mechanism in determining the type of cell death was further validated by in vitro investigations using other p53-deficient cell lines than endometrial sarcoma cells such as PANC-1, Jurkat, HL-60, and U937.

This presumed inhibitory activity of functional wild-type p53 protein in SAHA-mediated autophagy in our study was found to be highly consistent with the previously discovered general role of cytoplasmic p53 as a central coordinator of autophagy in normal cells [296]. Thus, while stress-induced transactivating activity of nuclear p53 protein promotes autophagy in a positive manner, direct interactions of physiological levels of the cytoplasmic p53 protein were found to block the induction of autophagy under normal conditions. Nevertheless, although this suppressive activity of p53 in the cytoplasm is obviously independent of its transcriptional function it engages the same canonical AMPK–mTOR signaling pathway cascade as nuclear p53. This mechanism entails, however, the inhibition of the AMP-dependent kinase and consequently the activation of mTOR signaling representing an opposed mechanism to nuclear-localized p53. By mutational analyses so far, direct interaction of p53 with FIP200 (ATG17) could be identified in this respect [297]. Pharmacological inhibition of basal levels of p53, as well as several genetically modified p53 mutants including a nuclear export domain-deficient form supported this newly identified mechanism of cytoplasm-controlled inhibition of autophagy. While these loss-of-function mutants of p53 activated autophagy, several variants with gain-of-function mutations could still exert a suppressive function on autophagy. From the physiological point of view, mutant p53 protein presumably enforces cell survival and provides increased metabolic stress resistance by activating cytoplasm-induced autophagy as it cannot keep its tumor suppressor function upright in the cell. This finding stresses again the importance to discriminate p53 variants with respect to their specific mutations, particular when considering that, as in our case, nonsense mutations can also provoke the degradation of the p53 transcript.

Further negative regulation of autophagy involving cytoplasmic p53 has also been demonstrated in embryonal carcinoma cells, where its interaction with Beclin-1 causes ubiquitination and degradation of the protein [298]. Functional inactivation of p53 therefore induces autophagy in these cells. Here, USP10 and USP13 ubiquitin-specific peptidases could be elaborated as mediators of de-ubiquitination activity of p53 [298]. Also, this mechanism of Beclin-1-induced autophagy needs to be further clarified in detail, however. Importantly, this type of autophagy involves the regulation of BCL2-family members and can be suppressed by caspase-mediated cleavage of Beclin-1; by concurrent production of Beclin-1 fragments, apoptosis can be activated by mitochondrial cytochrome C release. The use of a BCL-2 family inhibitor (GX15-070 or knockdown of BCL-2, BCL-XL, and MCL-1) in pancreatic cancer cells treated with SAHA or sodium valproate and sorafenib therefore induced autophagy and intrinsic apoptosis in another study accordingly, thereby helping to overcome a blockade of extrinsic apoptosis [299]. Consistently, p53-regulated pro-apoptotic proteins, such as PUMA, BAX, BNIP3, and BAD, have been identified as autophagy-inducing proteins [300,301]. Vice versa, a strategy combining HDACi that upregulate BIM expression and BH3 mimetics that release BIM from anti-apoptotic proteins, an event normally mediated by p53, has been shown to provide a link for activating apoptosis and disabling cytoprotective autophagy [302,303]. Furthermore, calpain-induced generation of an ATG5 fragment was reported to induce apoptosis [304]. If confirmed in vivo, mechanisms involving crosstalk between apoptosis and autophagy that comprise commonly regulated factors of both pathways would perfectly fit cells where activation of HDACi-induced apoptosis and/or autophagy occurs. Both cell death pathways could be regulated by common factors, and each could regulate and modify the activity of the other.

## 9. Further Evidence of HDACi-Mediated Apoptosis and Autophagy Regulation

Besides our report, HDACi-dependent autophagic induction involving p53 was also specified in four reports as a molecular cause of autophagic cell death strengthening the idea that p53 acts as a major regulator of HDACi-induced cell fate (see Table 1) [279,290,305,306]. Similar to our study, inhibition of p53-deficient pancreatic cancer cells by HDACi VPA and TSA was found to result in induction of apoptosis and autophagy in a recent report [279]. HDACi-mediated cell death induction was thereby found to correlate with their ability to reduce mutant p53 expression and to inhibit ERK phosphorylation as well as c-MYC expression presumably via acetylation; ERK-mediated stabilization of the oncogenic protein c-MYC has been previously known to activate cell proliferation and mediate a pro-survival pathway in cancer cells. In contrast to our study of endometrial stroma carcinoma cells that were completely devoid of p53 and where autophagy was predominantly upregulated, reactivation of wild-type p53 occurred in VPA and TSA-treated pancreatic cancer cells. This was furthermore accompanied by upregulation of the p53-induced target proteins p21 and PUMA. This is also likely to explain the concomitant upregulation of apoptosis and autophagy in these cells opposite to our findings where apoptosis was downregulated and could only be re-activated following reconstitution of p53. Intriguingly, reduced induction of autophagic cell death mediated by TSA in Panc1 cells furthermore correlated with a lack of Mcl-1 reduction and of ROS production compared to PaCa44 cells. In addition, this report consistently reflects the previously documented response of HDACi in suppressing mutant p53 that exhibits a dominant-negative effect and the associated re-induction of p53 wild-type expression as already detailed in chapter 7.

Sirtinol, a specific inhibitor of class III NAD-dependent deacetylases SIRT1 and SIRT2, has previously been associated with regulatory functions for mitosis, survival, and senescence by targeting p53. Increased acetylation of p53 following sirtinol treatment resulted besides other antiproliferative effects, such as cell cycle arrest and apoptosis, also in increased LC3-II expression and autophagy in MCF-7 breast cancer cells [290]. p53 was thereby demonstrated to regulate the balance between apoptosis and autophagy, as suppression of autophagy by 3-methyladenine led to an increase in apoptotic cell death in these cells documented by increased BAX expression, decreased BCL-2 protein accumulation, and by cytochrome C release. Furthermore, a combination of sirtinol and MHY2256 led similarly to induction of cell cycle arrest, apoptosis, as well as autophagy in MCF-7 cells [305]. MHY2256 is a novel strong inhibitor of SIRT1 enzyme activity and also suppresses the expression of SIRT1, 2, and 3 protein levels. Its activity was found to cause increased p53 activity by suppressing SIRT1-mediated acetylation of p53 at lysine 382, thereby preventing its degradation by MDM2 ubiquitination. A more direct role for SIRT1 in the regulation of autophagy was subsequently elaborated in mouse embryonic fibroblasts with a homozygous SIRT1 deletion which have been used for rescue experiments with either the *SIRT1* wild-type gene or a deacetylase-inactive mutant of *SIRT1* [256]. Restoration of autophagy under starvation conditions was only possible in fibroblasts harboring the *SIRT1* wild-type gene. Acetylation of the autophagic proteins ATG5, ATG7, and ATG8 were significantly elevated in SIRT1-deficient cells defining them as possible targets of SIRT1-mediated deacetylation activity. The anticancer activity of MHY2256 was also investigated in Ishikawa cells derived from an endometrial cancer with a poor prognosis [306]. Also, in Ishikawa cells, MHY2256 induces apoptosis and autophagic cell death via p53 regulation. MHY2256 significantly increased acetylation and expression levels of p53, thereby inhibiting binding and downregulating the expression of its negative regulator, MDM2. MHY2256 was documented to sensitize Ishikawa cancer cells to apoptosis by increased Bax expression, cytochrome C release, upregulated expression of cleaved PARP, and slightly elevated Bcl-2 expression. G1 phase arrest induced by p21 upregulation may also contribute to the increased late apoptosis caused by MHY2256 in these cells. Increased autophagy in Ishikawa cells was found to contribute to highly apoptotic cytotoxicity of MHY2256. Nevertheless, a further study also noted HDACi-promoted cell death type-I and –II irrespective of p53 presence or absence [307]. As the p53 mutational status in apicidin-treated YD-8 and YD-10B human oral squameous carcinoma cells (OSCC) that underwent apoptosis and autophagy was different, it was concluded that induction of cell death and cell cycle arrest by upregulation of p21WAF1 occurred in a p53-independent manner.

Previous evidence also demonstrates that HDACi induce cancer cells to undergo autophagy and apoptosis by activating ROS [234,250,308,309]. One of these studies clearly demonstrates that the ROS-activated p38 MAPK/ERK-Akt cascade acts as a switch between cell death type-I and -II induced by MS-275 in HCT116 colon cancer cells [308]. Mitogen activated protein kinase (MAPK) signaling pathways activated by oxidative stress and other external stimuli are responsible for regulating a variety of cellular activities, including proliferation, differentiation, and apoptosis. High expression levels of p38 MAPK induced autophagy, while low expression levels activated apoptosis which was correlated with the duration of HDACi treatment. ROS-induced ERK activation, mediated by p38 MAPK, triggered *ATG7* expression and the induction of autophagy in short-term MS-275-treated HCT116 cells. However, after prolonged treatment above 48 h or silencing of *ATG7*, the p38 MAPK-activated pathway shifted towards activation of apoptosis. This time point also coincided with a decrease of high levels of phosphorylated ERK and the accumulation of phosphorylated JNK and AKT proteins. ROS accumulation has furthermore been detected in three other reports, two of them showing simultaneous activation of apoptosis and autophagy, while in one study autophagy was suppressed [234,250,309]. Many studies including HDACi-induced apoptosis and autophagy also detected increased acetylation of the core histones H3 and H4, confirming the basic activity of the respective HDACi, p21 upregulation, mTOR inactivation, and the involvement of pro- or anti-apoptotic proteins (see Table 1). Augmented acetylation of histones H3 and H4 has been consistently reported after HDACi treatment, and has been associated with increased transcription of distinct genes implicated in tumor growth suppression [45,313]. Therefore, measuring the grade of H3 and H4 histones acetylation during HDACi treatment is employed to assure their inhibitory effect [314]. A molecular switch between apoptosis and autophagy was, however, not specified in these reports.

## 10. Conclusions and Future Perspectives

In summary, our data support a master regulatory role for p53 with regard to SAHA and potentially also HDACi-mediated cell death in general [231,315]. Considering the accumulated knowledge about the molecular effects of HDACi on p53 function, the mutational status of p53 could give important information about a favorable or adverse response towards HDACi treatment in cancer therapy. Also, with regard to the controversial issue whether HDACi exert a cytoprotective or cytotoxic role in tumor cells and promote either apoptosis or autophagy, different tumor-specific alterations such as mutant p53 variants could provide an explanation. Nevertheless, future experiments need to explore the exact underlying molecular mechanisms that were found in the direct interference of SAHA with HDAC activity responsible for deacetylating the non-histone protein p53. As we previously detailed, with persistent overexpression of HDAC2, a class II enzyme, in malignant endometrial stromal sarcoma, it is possible that SAHA suppresses its direct deacetylating activity of p53, thereby activating apoptosis in ESS-1 (and MES-SA) cells [295,316]. However, when p53 is absent in the cell, the same mechanism demands the induction of autophagy, possibly by interfering with the deacetylation activity of autophagic regulatory proteins. This study highlights again the pivotal importance to address the context-specific function of the oncogenic tumor suppressor p53 in promoting or impeding autophagy before cytotoxic drugs should be applied for tumor therapy. Our findings also imply that in contrast to cases where inhibition of autophagy in addition to HDACi treatment was found to enhance anticancer effects, additional activation or stimulation of autophagy could help to overcome frequently encountered tumor cell-specific mutant p53 oncogenic activity and the associated apoptosis-resistance.

## Figures and Tables

**Table 1:** Overview of histone deacetylase inhibitor-triggered apoptotic and autophagic cell death.

HDACi ^1^	Cell Type	Apoptosis	Autophagy	Mechanism	Ref.
SAHA ^2^	Uterine sarcoma cell line (ESS-1)	Up	° Down	p53-deficiency; PUMA ↓ p21, CASP-9, -3, -7 ↑; mTOR, LC3, MDC ↓	[18]
VPA ^3^, TSA ^4^	Pancreatic cancer cells (PaCa44, Panc1)	Up	Up	Mutant p53 & c-Myc expr. ↓ ERK activ. ↑ p21, PUMA, Sub-G1, Bim, Bax, Bak, cyt. C, CASP3, AnnV ↑ Mcl-1 ↓ ROS ↑, p62 ↓, LC3-II ↑	[279]
Sirtinol	MCF-7	* Up	Up	SIRT1, SIRT2, SIRT3 ↓; p53 acetyl. ↑; Sub-G1, Bax, cyt. C, AnnV ↑ Bcl-2 ↓; LC3-II, AVO, MDC ↑	[290]
MHY2256	MCF-7, (SKOV-3)	Up	Up	SIRT1, 2, 3 expr. & SIRT1 activity ↓; p53 acetyl. ↑ MDM ↓; p21 ↑ Annexin V/PI,, Bax ↑, Bcl-2 ↓, PARP cleavage ↑ LC3-II, ATG5, AVO ↑	[305]
MHY2256	Ishikawa endometrial cancer cells	Up	Up	SIRT1, 2, 3 expr. & SIRT1 activity ↓; p53 acetyl. ↑ MDM ↓; p21 ↑ Annexin V/PI, Bax, Bcl-2, PARP cleavage ↑ LC3-II, ATG5, AVO ↑	[306]
Apicidin	OSCC (YD-8, YD-10B)	* Up	Up	H3 & H4 acetyl.↑; p53, CycB1 ↓; p21, LC3-II, ATG5, AVO ↑	[307]
MS-275	HCT116	Up	Up	P38 MAPK ↓ ROS > 48 h; CASP-8, -3, -9, PARP ↑; P38 MAPK; ROS < 48 h, ERK, ATG7 expr., LC3-II ↑	[308]
SAHA	Jurkat T-cells	* Up	Up	TUNEL+- cells ↑; ROS ↑, mTOR ↓; BECN1, ATG7, ATG12-5, LC3-II, AVO ↑	[234]
VPA,SAHA	AML cells (Kasumi-1)	* Up	Up	ROS ↑; CASP3, PARP cleavage ↑; LC3-II, LC3 staining ↑	[309]
SAHA, TSA, VPA, MS-275, JQ2	DS-AMKL cells	Up	Down	H3 & H4 acetyl. ↑; HDAC1 & 2 inhibition; ROS ↑ Ann V/7-AAD ↑; 409 autophagic proteins, ATG7, LC3-II ↓	[250]
PCI-24781, SAHA, MS-275	MPNST cell lines	* Up	Up	H3, H4, tubulin acetyl. ↑ Ann. V/PI, PARP cleavage ↑; AVO staining, LC3-II, IRGM, CXCR4, TMEM74 ↑ Nf-κB ↓	[292]
FK228	MRT cells	* Up	+ Up	H3 & H4 acetyl. ↑; CASP & AIF translocat. ↑; LC3-II ↑	[246]
MHY218	Tamoxifen- resistant MCF-7	Up	Up	H3 & H4 acetyl. ↑; HDAC1, -4, -6 expr. ↓ Annexin V/PI staining ↑ BECN1, LC3-II ↑	[310]
TSA	Neuroblastoma cells	Up	+ Up	H3 & H4 acetyl. ↑; p21↑; Bax, Bid, Bcl-2, surviving ↓ PARP, CASP3↑; BNIP3, LC3-II ↑	[311]
SAHA	Chondrosarcoma (SW1353, RCS, OUMS-27) cells	Up	Up	H3 acetyl. ↑; Sub-G1, PARP cleavage ↑ LC3-II ↑	[284]
H40, SAHA	PC3-M, HL-60	Up	Up	H3 acetyl.; p21 ↑ Annexin V/PI, MDC ↑	[244]
LAQ824, LBH589	Eu-myc lymphoma	Up	+ Up	Bcl-XL dependent intrinsic apoptosis ↑ inhibition by Bcl-2/Bcl-X overexpr.; LC3-II; morph. change ↑	[249]
MGCD0103	CLL cells	Up	Down	Intrinsic apoptosis ↑; PI3K/AKT/mTOR & CAPN1 ↑; HDAC6, DRAM1; ATG7 & 12 ↓	[238,312]
SAHA	Glioblastoma cells (T98G)	* Up	+ Up	Caspase-3 ↑; mTOR inactivation ↑; ULK-1 activation, ATG7, LC3 ↑	[233]
Butyrate, SAHA	HeLa, (SKOV-3, U251)	Up	Up	Intrinsic apoptosis ↑ cyt. C, CASP-3 ↑ Autophagic Morphology ↑	[222]
SAHA	Glioblastoma stem cells	* Up	Up	CASP-3, PARP cleavage ↑ mTOR, p62/SQSTM1↓ BECN1 LC3-II, AVO ↑	[235]

^1^ HDACi, histone deacetylase inhibitor; ^2^ SAHA, suberoylanilide hydroxamic acid; ^3^ VPA, valproic acid; ^4^ TSA, trichostatin A * Increased apoptosis or + autophagy following inhibition of autophagy or apoptosis, respectively; ° Can be reactivated by wild-type p53 reconstitution; ↑ upregulation or activation; ↓ downregulation or inhibition; OSCC, oral squamous carcinoma cells; CLL, chronic lymphocytic leukemia; AVO, acidic vesicular organelles detected by acridine-orange staining; CAPN1, calpain-1; MRT, malignant rhabdoid tumor; PI, propidium iodide; MDC, monodansylcadaverine (staining); MPNST, malignant peripheral nerve sheath tumors; MDM2, mouse double minute 2; DS-AMKL, down syndrome associated myeloid leukemia; AML, acute myeloid leukemia.
